# Comparison of corneal irregular astigmatism by the type of corneal regular astigmatism

**DOI:** 10.1038/s41598-021-95358-z

**Published:** 2021-08-04

**Authors:** Yuta Ueno, Risa Nomura, Takahiro Hiraoka, Katsuhito Kinoshita, Mutsuko Ohara, Tetsuro Oshika

**Affiliations:** 1 Department of Ophthalmology, Faculty of Medicine, University of Tsukuba, 1-1-1 Tennoudai, Tsukuba, Ibaraki 305-8575 Japan; 2 Ito Eye Clinic, Ibaraki, Japan

**Keywords:** Biomarkers, Medical research, Pathogenesis

## Abstract

We investigated the relation between corneal regular and irregular astigmatism in normal human eyes. In 951 eyes of 951 patients, corneal irregular astigmatism, such as asymmetry and higher-order irregularity components, was calculated using the Fourier harmonic analysis of corneal topography data within the central 3-mm zone of the anterior corneal surface. The eyes were classified by the type of corneal regular astigmatism into four groups; minimum (< 0.75 diopters), with-the-rule (WTR), against-the-rule (ATR), and oblique astigmatism. The mean age was significantly different among the four groups (*P* < 0.001); patients with WTR astigmatism were the youngest, followed by those with minimum, oblique, and ATR astigmatism. Significant inter-group differences were found among the four groups in asymmetry (*P* = 0.005) and higher-order irregularity components (*P* < 0.001); the largest was in eyes with oblique astigmatism, followed by ATR, WTR, and minimum astigmatism. The stepwise multiple regression analysis revealed that corneal regular astigmatism pattern significantly influenced the amount of corneal irregular astigmatism after controlling for confounding factors (*P* < 0.001). Corneal irregular astigmatism, such as asymmetry and higher order irregularity components, was the largest in eyes with oblique astigmatism, followed by those with ATR, WTR, and minimum astigmatism, even after adjustment for age of subjects.

## Introduction

The cornea has two forms of astigmatism; regular and irregular astigmatism. Among them, irregular astigmatism is defined as astigmatism that cannot be corrected with spherocylindrical spectacle lenses, and thus is one of the important causes of suboptimal visual function, such as corrected visual acuity and contrast sensitivity, in otherwise healthy eyes^1, 2^.

 Pathological changes in corneal irregular astigmatism can be induced by various kind of corneal diseases^3–8^ as well as ocular surgeries^9–14^. Physiologically, corneal irregular astigmatism in normal human eyes is known to increase along with aging^15–21^. The relation between corneal regular and irregular astigmatism, however, has not been studied in detail, and it is yet to be clarified whether there is any association between corneal irregular astigmatism and the type of corneal regular astigmatism, such as with-the-rule (WTR), against-the-rule (ATR), and oblique astigmatism. Previous studies^22–24^ have shown that eyes with oblique astigmatism showed worse visual functions than those with ATR astigmatism, and that eyes with WTR astigmatism were least affected. After monofocal intraocular lens implantation, uncorrected distance visual acuity (UDVA) in eyes with simple myopic ATR was worse than in eyes with simple myopic WTR astigmatism^25^. It is, therefore, of clinical as well as pathophysiological interest to assess corneal irregular astigmatism in normal human eyes in relation to the pattern of corneal regular astigmatism.

## Patients and methods

### Patients

We retrospectively collected the data of patients who had undergone ocular examinations at Tsukuba University Hospital or Ito Eye Clinic. Patients were selected from the consecutive cohort according to the following criteria. Eyes were excluded from the study if they had ocular diseases except for mild to moderate cataract, any history of eye surgery including cataract surgery, or use of a contact lens within 3 weeks. Eyes with advanced cataract were excluded due to possible poor visual fixation during measurements.

### Examinations

The eyes were examined with swept-source anterior segment optical coherence tomography (AS-OCT) (CASIA, SS-1000, Tomey, Nagoya, Japan), which is a three-dimensional Fourier domain OCT using a 1,310-nm light source. Its axial and transverse resolutions are 10 µm and 30 µm, respectively. The device uses auto alignment and auto focus of the examined eyes. The scanning speed is 30,000 A-scans per second, and the acquisition time is ≤ 0.3 s. Its accuracy and reliability of corneal measurements have been reported elsewhere^26, 27^. The quality of AS-OCT images was confirmed by the examiner after each measurement before storage.

From the anterior corneal curvature data, regular astigmatism was calculated using the keratometric index. Based on corneal regular astigmatism, the eyes were classified into four groups; minimum astigmatism (< 0.75 diopters, D), WTR astigmatism, ATR astigmatism, and oblique astigmatism groups. When the steepest meridian of the anterior cornea was within ± 30 degrees of the vertical axis, the astigmatism was judged to be WTR. Those with the steepest meridian of ± 30 degrees of the horizontal axis were considered to have ATR astigmatism. All others were regarded as oblique astigmatism.

For the calculation of irregular astigmatism, anterior corneal dioptric data of AS-OCT were expanded into spherical power, asymmetry component (first order harmonic), regular astigmatism (second order harmonic), and higher-order irregularity component (third and higher order harmonic) using Fourier harmonic analysis^28^. Asymmetry and higher-order irregularity components computed within the central 3-mm zone were used to represent corneal irregular astigmatism.

The study protocol was reviewed and approved by the Institutional Review Board of University of Tsukuba. This study was conducted in accordance with the Declaration of Helsinki. The informed consent was obtained from each patient.

### Statistical analysis

For assessment of data among four groups, the multiple comparison test was employed, i.e. one-way analysis of variance (ANOVA) with Bonferroni correction. The stepwise multiple regression analysis was performed to evaluate the influence of age and type of regular astigmatism (explanatory variables) on the amount of irregular astigmatism (dependent variable). The type of regular astigmatism was converted to dummy variables, such as (1, 0, 0, 0), (0, 1, 0, 0), (0, 0, 1, 0), and (0, 0, 0, 1). All statistical tests were 2-sided and a p-value of less than 0.05 was considered significant. The numerical data are presented as the mean ± standard deviation (SD) unless otherwise noted. Statistical analysis was performed using SPSS Statistics for Windows software (version 26, IBM Corp., Armonk, NY, USA).

### Results

**Figure 1:**
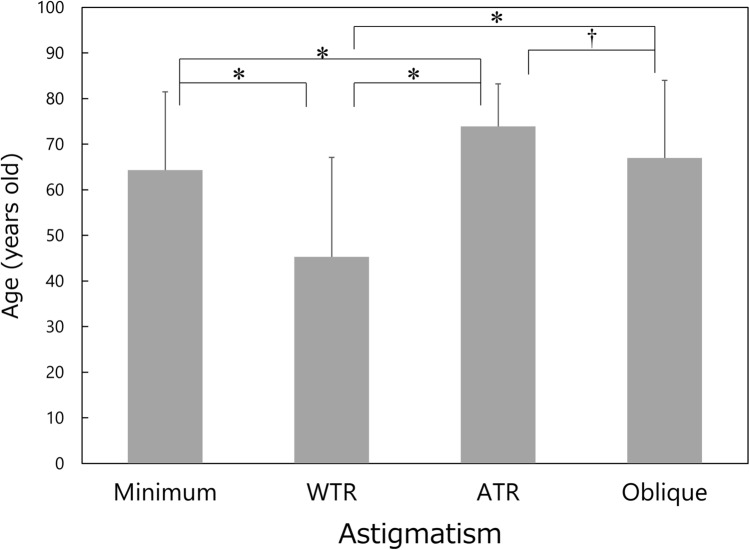
Comparison of age among the four types of corneal regular astigmatism groups. Mean ± standard deviation, *WTR* with-the-rule, *ATR* against-the-rule, *P < 0.001, †*P* = 0.010.

Data from 951 eyes of 951 patients were collected and analysed. There were 449 males and 502 females, and their age averaged 60.9 ± 20.1 (range 6–93) years old. The background data of patients are shown in Table 1. There was a significant difference in the mean age among groups (*P* < 0.001), and the post-hoc test revealed significant differences between each group except for between minimum and oblique astigmatism groups (Fig. 1). The patients with WTR astigmatism were the youngest, followed by those with minimum, oblique, and ATR astigmatism.

**Table 1:** Background data of patients.

	Type of corneal regular astigmatism			
	Minimum (< 0.75 D)	WTR	ATR	Oblique
Number of eyes	350	334	194	73
Male/female	157/193	161/173	99/95	32/41
Age (years old)	65.9 ± 15.1 (7–88)	47.0 ± 22.0 (6–90)	73.8 ± 8.5 (41–93)	66.4 ± 17.4 (8–89)

D: diopter, *WTR* with-the-rule, *ATR* against-the-rule, mean ± standard deviation (range).

**Figure 2:**
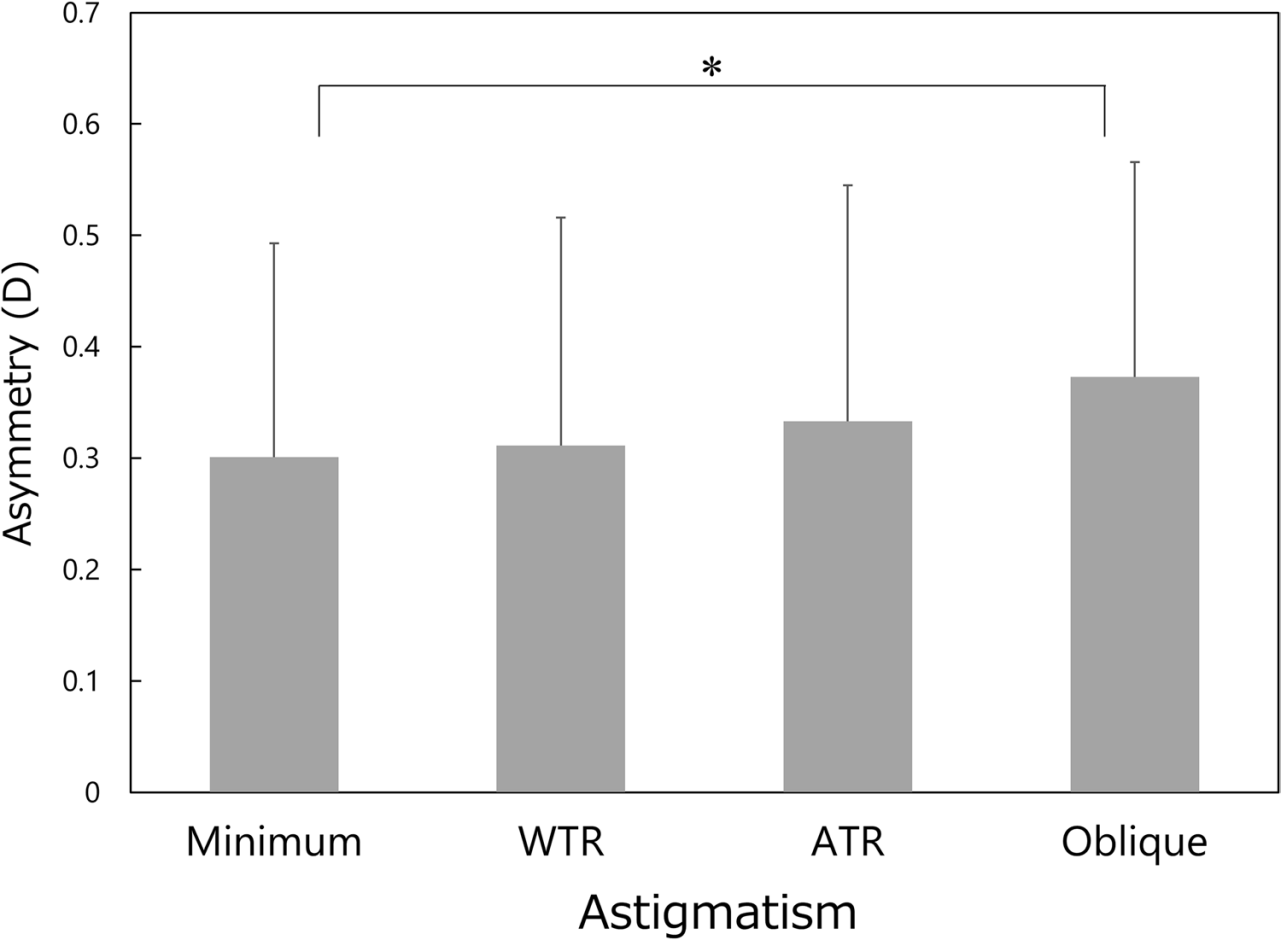
Comparison of asymmetry component among the four types of corneal regular astigmatism groups. Mean ± standard deviation, *WTR* with-the-rule, *ATR* against-the-rule, *P = 0.050.

**Figure 3:**
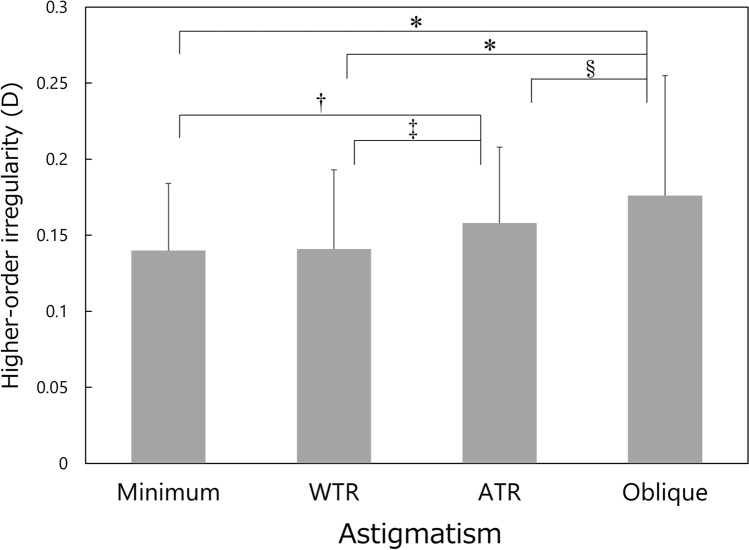
Comparison of higher-order irregularity component among the four types of corneal regular astigmatism groups. Mean ± standard deviation, *WTR* with-the-rule, *ATR* against-the-rule, *P < 0.001, †*P* = 0.009, ‡*P* = 0.044, §*P* = 0.015.

Significant differences were found among the four groups in the amount of corneal irregular astigmatism, such as asymmetry (*P* = 0.005) and higher-order irregularity components (*P* < 0.001). The asymmetry component was significantly smaller in the minimum astigmatism group than in the oblique astigmatism groups (*P* = 0.050), while statistically significant differences were not found between other groups (Fig. 2). In the higher-order irregularity component, significant inter-group differences were found for every match-up (Fig. 3), except for between the minimum and WTR astigmatism groups (*P* = 1.000).

In all 951 eyes, the amount of regular astigmatism significantly correlated with those of asymmetry (r = 0.092, *P* = 0.004) and higher-order irregularity (r = 0.164, *P* < 0.004). In eyes with minimum astigmatism, regular astigmatism did not correlate with asymmetry (r = − 0.015, *P* = 0.402) and higher-order irregularity (r = 0.075, *P* = 0.160). In eyes with WTR astigmatism, regular astigmatism significantly correlated with asymmetry (r = 0.155, *P* = 0.004) and higher-order irregularity (r = 0.162, *P* = 0.003). In eyes with ATR astigmatism, regular astigmatism did not correlate with asymmetry (r = − 0.028, *P* = 0.694) and higher-order irregularity (r = 0.0782 *P* = 0.225). In eyes with oblique astigmatism, regular astigmatism did not correlate with asymmetry (r = 0.055, *P* = 0.643), but significantly correlated with higher-order irregularity (r = 0.534, *P* < 0.001).

The stepwise multiple regression analysis revealed that age (partial correlation coefficient r = 0.258, *P* < 0.001) and type of regular astigmatism (WTR astigmatism r = 0.157, *P* < 0.001, oblique astigmatism r = 0.082, *P* = 0.011) were significantly associated with the asymmetry component. Parameters that were significantly relevant to the higher-order irregularity component included age (r = 0.161, *P* < 0.001) and type of regular astigmatism (minimum astigmatism r = − 0.097, *P* = 0.003, oblique astigmatism r = 0.129, *P* < 0.001).

## Discussion

In general, corneal irregular astigmatism, including higher-order aberrations, increases along with aging in normal human eyes^15–21^. In the current study, the multiple regression analysis indicated that age is one of the important factors that contribute to the increases of corneal irregular astigmatism. When comparing the age of each group (Fig. 1), subjects in the ATR astigmatism group were the oldest, followed by those in the oblique, minimum, and WTR astigmatism groups. This pattern (order of four groups) is different from that of corneal irregular astigmatism, including asymmetry (Fig. 2) and higher-order irregularity component (Fig. 3), in which the parameters were the largest in eyes with oblique astigmatism, followed by those with ATR, WTR, and minimum astigmatism. In addition, the stepwise multiple regression revealed that the type of corneal regular astigmatism significantly contributes to the degree of corneal irregular astigmatism, after adjustment for age. These results indicate that corneal irregular astigmatism is influenced by the type of corneal regular astigmatism, independently of the age of subjects.

To the best of our knowledge, the current study represents the first report on the association between corneal irregular astigmatism and the type of corneal regular astigmatism, by controlling for confounding factors. In a previous study, Kiuchi et al.^29^ evaluated 426 patients with AS-OCT and reported that age and the amount of corneal regular astigmatism showed significant correlations with corneal higher-order aberrations, whereas the amount of spherical equivalent and refractive status (myopia, emmetropia, or hyperopia) did not. Choi et al.^30^ investigated the changes in corneal higher-order aberration during amblyopia treatment, and assessed the correlation between higher-order aberration and astigmatism in hyperopic amblyopia children. They found that coma higher-order aberration significantly correlated with astigmatism and could exert effects in cases involving hyperopic amblyopia. Cheng et al.^31^ examined the relationship between ametropia and optical aberrations in a population of 200 normal human eyes with refractive errors spanning the range from + 5.00 to − 10.00 D, and reported that astigmatic eyes tended to have larger total higher-order aberrations than nonastigmatic eyes. Leung et al.^32^ evaluated the corneal shapes and monochromatic aberrations in Chinese myopic adults with and without astigmatism, and demonstrated significant relationships between astigmatism, corneal shapes, and monochromatic aberrations. Compared with simple myopia, myopic astigmatism had more oblate nasal and temporal corneal shapes and showed significantly more negative Y trefoil and more positive vertical coma. The asymmetry in corneal shape along the vertical principal meridian (inferior—superior) was significantly associated with the Y trefoil and vertical coma of the cornea, suggesting that this regional asymmetry in corneal shape may contribute to the ocular aberrations. Our study indicated that eyes with oblique astigmatism is associated with the greatest amount of asymmetry component as well as high-order irregularity component, in line with the findings of aforementioned studies.

With a greater amount of corneal irregular astigmatism, optical quality of eyes with oblique astigmatism may be deteriorated to some extent. The influence of axis orientation of uncorrected astigmatism on visual function has been studied previously in normal eyes of healthy volunteers^22–24^ and eyes after monofocal intraocular lens implantation^25^. The studies in volunteers reported that oblique astigmatism induced with cylindrical lenses resulted in worse visual functions than ATR astigmatism, and that WTR astigmatism had the least effect^22–24^. In eyes after intraocular lens implantation, it was demonstrated that UDVA was worse in eyes with simple myopic ATR astigmatism than in those with simple myopic WTR astigmatism^25^. These studies, however, were about the influence of uncorrected corneal regular astigmatism on visual function. In order to elucidate the impact of naturally occurring corneal irregular astigmatism as in our study, the relation between spectacle-corrected visual functions and type of corneal regular astigmatism should be investigated, though such studies are not available until now. Further studies are awaited.

The present study has several limitations. First, being a retrospective study, some important data are missing, such as axial length and refractive status. Previous studies, however, showed that refractive status (myopia, emmetropia, or hyperopia) is not correlated with corneal higher-order aberrations when age is taken into account^23, 25^, and thus we believe that our study is not largely biased by the absence of those data. Other factors, such as intraocular pressure, anterior chamber depth, corneal thickness, and corneal diameter were not analyzed in this study, which would be the subject of future studies. Second, we did not analyze the impact of corneal irregular astigmatism on visual function, such as visual acuity and contrast sensitivity. Thus, it is not clear to what extent the current findings have clinical relevance. Nonetheless, the current results indicate that eyes with oblique astigmatism are associated with deteriorated optical quality, where consideration is needed in the indication of toric intraocular lenses, selection of hard/soft contact lenses, and performance of surgical correction of astigmatism on the cornea. Third, we presented the data obtained from the measurements of corneal anterior surface alone, and the influence of posterior corneal astigmatism was not considered. Since the astigmatism pattern is totally different between the anterior and posterior corneal surfaces^34–35^, it seems more desirable to analyze the total corneal astigmatism by combining both anterior and posterior corneal astigmatism. These are the subjects of future studies.

In conclusion, we investigated the relation between corneal regular and irregular astigmatism. Corneal irregular astigmatism was calculated using Fourier harmonic analysis of corneal topography data obtained with AS-OCT, and was analyzed according to the type of corneal regular astigmatism; minimum (< 0.75 D), WTR, ATR, and oblique astigmatism. It was found that corneal irregular astigmatism, such as asymmetry and higher order irregularity components, was the largest in eyes with oblique astigmatism, followed by those with ATR, WTR, and minimum astigmatism, even after adjustment for age of subjects.
